# A Model Transfer Method among Spectrometers Based on Improved Deep Autoencoder for Concentration Determination of Heavy Metal Ions by UV-Vis Spectra

**DOI:** 10.3390/s23063076

**Published:** 2023-03-13

**Authors:** Hongqiu Zhu, Yi Shang, Qilong Wan, Fei Cheng, Haonan Hu, Tiebin Wu

**Affiliations:** 1 School of Automation, Central South University, Changsha 410083, China; 2 School of Energy and Electromechanical Engineering, Hunan University of Humanities, Science and Technology, Loudi 417000, China

**Keywords:** spectrometry, micro-spectrometer, model transfer, autoencoder, heavy metal ion

## Abstract

Ultraviolet Visible (UV-Vis) spectroscopy detection technology has been widely used in quantitative analysis for its advantages of rapid and non-destructive determination. However, the difference of optical hardware severely restricts the development of spectral technology. Model transfer is one of the effective methods to establish models on different instruments. Due to the high dimension and nonlinearity of spectral data, the existing methods cannot effectively extract the hidden differences in spectra of different spectrometers. Thus, based on the necessity of spectral calibration model transfer between the traditional large spectrometer and the micro-spectrometer, a novel model transfer method based on improved deep autoencoder is proposed to realize spectral reconstruction between different spectrometers. Firstly, two autoencoders are used to train the spectral data of the master and slave instrument, respectively. Then, the hidden variable constraint is added to enhance the feature representation of the autoencoder, which makes the two hidden variables equal. Combined with a Bayesian optimization algorithm for the objective function, the transfer accuracy coefficient is proposed to characterize the model transfer performance. The experimental results show that after model transfer, the spectrum of the slave spectrometer is basically coincident with the master spectrometer and the wavelength shift is eliminated. Compared with the two commonly used direct standardization (DS) and piecewise direct standardization (PDS) algorithms, the average transfer accuracy coefficient of the proposed method is improved by 45.11% and 22.38%, respectively, when there are nonlinear differences between different spectrometers.

## 1. Introduction

A UV-Visible spectrum instrument is a very important optical analysis detector to determine composition and substances concentration. A traditional spectrometer has good measuring accuracy and repeatability. However, it has obvious shortcomings such as large size, high price and a complex experimental process, which cannot meet the requirements of online detection and analysis. Compared with the traditional large spectrometer, the micro-spectrometer has the advantages of low price, miniaturization, simple maintenance and so on, which makes it possible for the spectral detection technology to be applied from the laboratory environment to the complex industrial field [1]. Under general conditions, the multivariate correction model established by the target value and spectral signal of the sample is usually used for the same spectrometer [2]. However, the measurement spectral differences caused by the hardware differences between different spectrometers will have a serious impact on the ion concentration prediction results, which causes the existing correction models to no longer be applicable [3].

Model transfer can reduce the interference on the spectrum of external interference and other factors, so as to ensure that the previously established model can continue to be applied and further avoid the consumption of manpower. Researchers have conducted a lot of research and summary on model transfer methods, including the direct standardization method (DS) [4,5], piecewise direct standardization method (PDS) [6,7] and machine learning-based method [8,9,10]. In recent years, the direct standardization and piecewise direct standardization method have been widely used in many fields [11,12,13,14]. Alves applied the piecewise direct standardization method to overcome matrix effects in front face fluorescence spectroscopy of a mixture when different excipients of acetylsalicylic acid, paracetamol and caffeine were used [15]. Zeng used the direct standardization algorithm to effectively correct the spectral changes of confocal micro-Raman spectrometers and portable Raman spectrometers in the laboratory [8]. Parrott proved that the direct standardization method can correct different spectral changes caused by changing the combination of spectrometer probes [16].

However, the direct standardization method and piecewise direct standardization method are used to build a linear relationship between instruments to achieve model transfer, which cannot solve the nonlinear differences between instruments. In recent years, with the rapid development of machine learning, more and more model transfer methods have been proposed by scholars. Zhang proposed a stability-analysis-based feature selection algorithm (SAFS) for near infrared (NIR) calibration transfer to tackle the variation of different spectrometers and environment change [17]. Chen proposed a new method that combines principal component analysis (PCA), weighted extreme value learning machine (ELM) and a TrAdaBoost algorithm [18]. The TrAdaBoost algorithm based on weighted ELM is applied to establish a series of calibration transfer quantitative analysis models. The transfer of a calibration model can be viewed as a missing data attribution problem. The trimmed scores regression (TSR) and joint-Y partial least squares regression (JYPLS) are used as a maximum likelihood estimation-principal component analysis to re-establish a transfer model [19]. The experimental results show that the method can greatly improve the prediction accuracy of the model. Galvao et al. use robust regression, employing univariate correction rather than the full spectrum or spectral windows for calibration model transfer [20]. The model result is satisfactory in the transfer of gasoline samples and corn samples with near infrared spectra of different spectrometers. Chen proposed a transfer model by an extreme learning machine auto-encoder method (TEAM) to correct the systematic differences between the spectra of different instruments successfully [21].

As more and more model transfer methods have been proposed by scholars, the existing model transfer algorithms have their own limitations. The use of full-spectrum modeling in the direct standardization method not only has a large amount of data and redundant interference information, but also greatly reduces the model efficiency. In addition, there is no adaptive method to select the window width by the piecewise direct standardization [22]. Since the spectral range and resolution measured by different types of spectral instruments are different, it is often difficult to search the direct correspondence wavelength points directly. In the case of high spectral data dimension and small sample size, it is easy to produce an overfitting problem.

For this reason, in view of the obvious spectral difference between the micro-spectrometer and the traditional large-scale spectrometer, a model transfer method based on an improved autoencoder is proposed to apply the model between different instruments. Firstly, the necessity of spectral model transfer is analyzed. Then, the constraint conditions of hidden variables are added based on an autoencoder. The hyper-parameters in the objective function are quickly optimized with a Bayesian optimization algorithm. The transfer accuracy coefficient is proposed to characterize the transfer performance of the model. Finally, the spectral data is reconstructed by training the transfer model. The experimental results show that the spectrum measured by the slave instrument basically coincides with the master spectrum after correction, and the phenomenon of wavelength point shift is also eliminated.

The rest of this paper is organized as follows. In Section 2, the proposed method is described in detail. The experimental design and comparative analysis are given in Section 3. Section 4 presents the conclusion of this paper.

## 2. Method

### 2.1. Fundamentals of Autoencoder Theory

**Figure 1:**
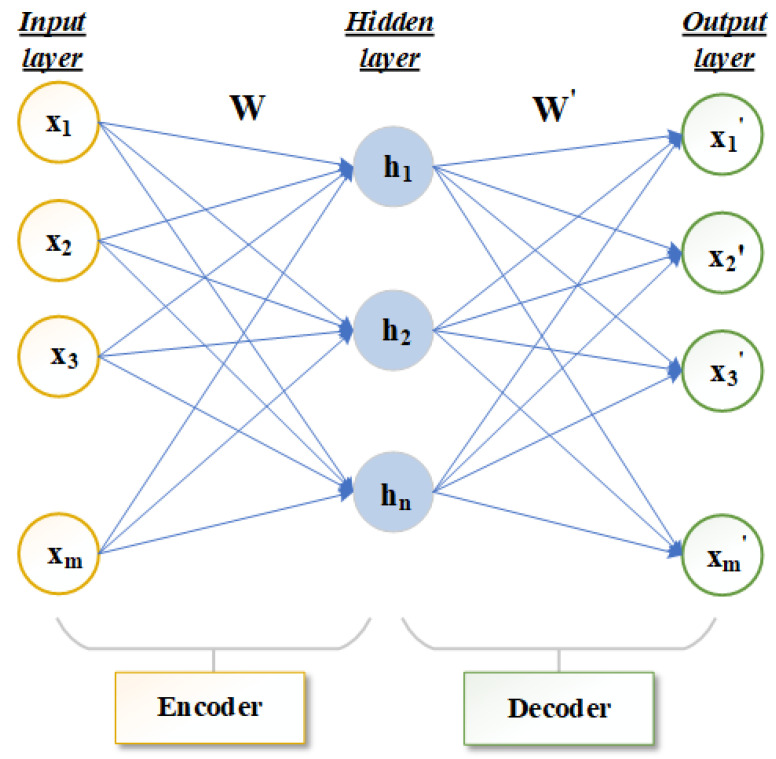
Structure diagram of autoencoder frame.

Autoencoder (AE) is a method of unsupervised deep learning [23,24]. It can express original data with complex features of high dimension into a more abstract data form of low dimension through a multi-layer neural network. Autoencoders are mainly used to automatically learn nonlinear data features and are widely used in effective coding, data dimension reduction and model generation [25]. The infrastructure of the autoencoder mainly includes the three-layer network structure of the input layer, hidden layer and output layer, as shown in Figure 1. The number of neurons in the input layer and output layer should be the same. If the number of neurons in the input layer and output layer is *m*, the number of neurons in the hidden layer is *n*, the input data is represented by $X=\{x_{1},x_{2},\ldots ,x_{m}\}$ and the hidden layer data is represented by $H=\{h_{1},h_{2},\ldots ,h_{n}\}$. The output layer is $X^{\prime }=\left\{x_{1}^{{}^{\prime }},x_{2}^{{}^{\prime }},\ldots ,x_{m}^{{}^{\prime }}\right\}$.

The main structure of an autoencoder is divided into encoder and decoder [26,27,28]. The network that transforms the high-dimensional data *X* of the input layer to the low-dimensional hidden layer in Figure 1 is called the encoder. The function *f* is the deterministic mapping. This process can be expressed in Equation (1):

(1) $$H=f\left(x\right)=s_{f}\left(WX+b\right)$$

where $s_{f}$ represents the activation function of the encoder. *W* is the mapping weight matrix of n×m dimension. $b\in R^{n}$ is a bias vector. The decoder is used to transform the hidden layer data *H* through function *g* to obtain the reconstructed output layer data $X^{\prime }$. This process can be expressed by Equation (2):

(2) $$X^{\prime }=g\left(H\right)=s_{g}\left(W^{\prime }H+b^{\prime }\right)$$

where $s_{g}$ represents the activation function of the decoder, and $W^{\prime }$ is the mapping weight matrix of m×n dimension. $b^{\prime }\in R^{m}$ is a bias vector. Encoder activation function $s_{f}$ and decoder activation function $s_{g}$ can perform well in feature learning by using a nonlinear activation function. The autoencoder continuously trains the input data *X* and $X^{\prime }$ to minimize the reconstruction error terms so that *X* and $X^{\prime }$ are as close as possible to achieve the best reconstruction results. Generally, the cross entropy calculation is used to reconstruct the error term, which can be expressed by Equation (3):

(3) $$E\left(X,X^{\prime }\right)=-\sum_{i=1}^{n}\left(x_{i}\log x_{i}^{{}^{\prime }}+\left(1-x_{i}\right)\log\left(1-x_{i}^{{}^{\prime }}\right)\right)$$

In general, the autoencoder describes the mapping between the high-dimensional data and low-dimensional data without losing the main information. Therefore, the number of neurons in the hidden layer of the autoencoder is smaller than the number of neurons in the input layer *m*.

### 2.2. Model Transfer Method Based on Improved Deep Autoencoder

In spectral model transfer, the basic idea is to make the spectrum measured from the instrument infinitely close to the spectral signal measured on the master instrument by constantly modifying the spectrum measured from the instrument under the same sample condition, so that these spectra are equivalent to being scanned from the master instrument. Then, a multivariate calibration model established on the master instrument is used to predict the corrected spectral data in order to minimize the difference in the detection results under different measurement conditions.

Data compression and decompression functions in autoencoders are data dependent and can be learned automatically from samples. According to this idea, the model transfer problem can be regarded as a process of data compression and decompression. In this paper, a model transfer algorithm based on an autoencoder is proposed to solve the problem that the spectral difference caused by different measurement conditions has a serious impact on the ion concentration prediction results.

**Figure 2:**
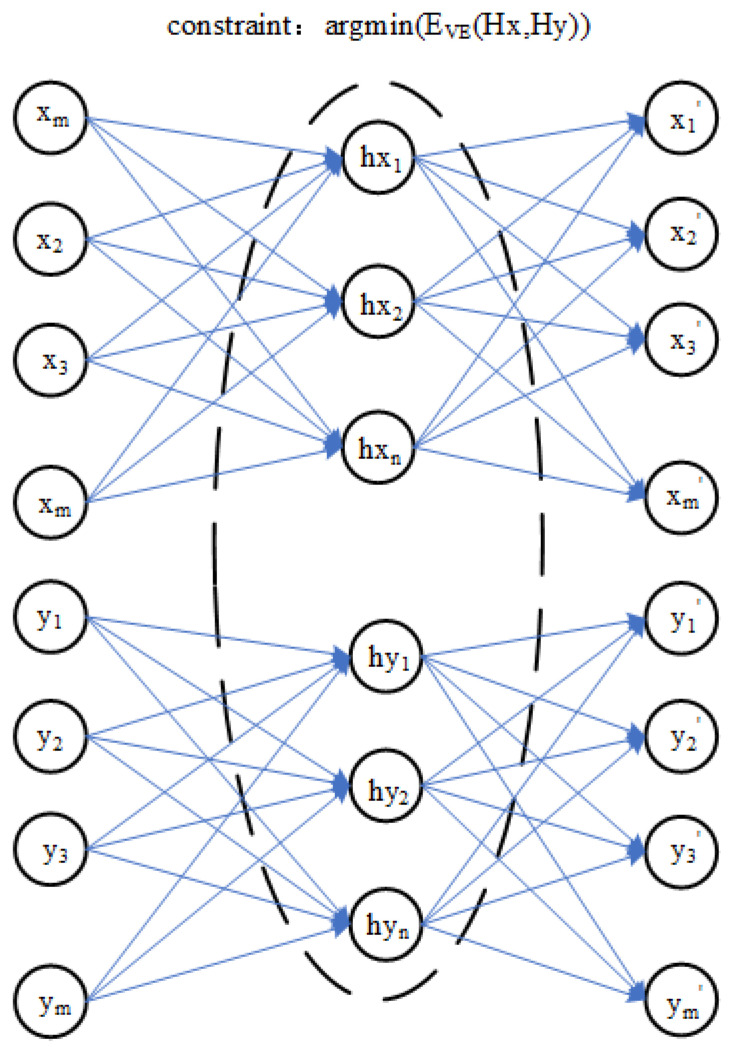
Training process of a model transfer algorithm based on a deep autoencoder.

A training model transfer algorithm based on the encoder and the optimization process is shown in Figure 2, which is based on the encoder model transfer algorithm of the parameter optimization process and can be decomposed into three parts:

In the first part, the master spectral data *X* and slave spectral data *Y* are used by the encoder to obtain hidden layer variables $H_{x}$ and $H_{y}$. The second part is to optimize the reconstruction error $E_{VE}\left(H_{x},H_{y}\right)$ between the hidden variables $H_{x}$ and $H_{y}$. In the third part, when the master spectral data *X* and slave spectral data *Y* obtain the reconstructed data $X^{\prime }$ and $Y^{\prime }$ through their respective autoencoders, the process is optimized by minimizing the reconstruction errors $E_{VE}\left(X,X^{\prime }\right)$ and $E_{VE}\left(Y,Y^{\prime }\right)$. Therefore, the objective function of the model transfer algorithm based on an autoencoder is proposed. The $E_{VE}\left(H_{x},H_{y}\right)$ error term of hidden layer variables $H_{x}$ and $H_{y}$ and the reconstruction error $E_{VE}\left(Y,Y^{\prime }\right)$ and $E_{VE}\left(X,X^{\prime }\right)$ are very important in the model transfer algorithm based on an autoencoder, which directly affects the quality of model training. In this paper, a polynomial likelihood function is used to calculate three error terms in the objective function of a model transfer algorithm of an autoencoder.

**Figure 3:**
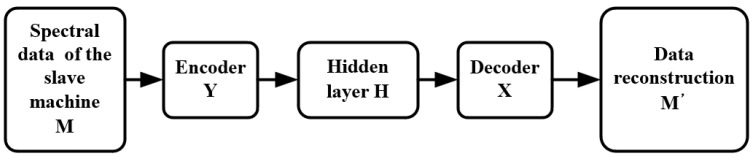
Training process of a model transfer algorithm based on a deep autoencoder.

Figure 3 shows the steps of model transfer. It is assumed that the hidden variable $H_{y}$ of the slave instrument is obtained from the spectral data *M* of the slave instrument through the encoder of the slave instrument. The final reconstructed data $M^{\prime }$ are obtained from $H_{y}$ through the decoder of the master instrument. The process of model transfer is completed based on an autoencoder.

In this paper, a Bayesian optimization algorithm is adopted for hyperparameter optimization to achieve optimal performance of the target network. In the process of hyperparameter exploration, it is necessary to master a balance: when searching in the upward direction of a definite curve, better calculation results can be obtained with a greater probability, but local optimal solutions can be easily obtained. In addition to the upward direction of the curve, other search areas should also be explored. This paper adopts the Probability of Improvement (PI) method based on a promotion strategy as a collection function to determine the next evaluation point. At last, the cross-validation method is used to select the optimal hyperparameters. In the process of selecting the optimal hyperparameters, the transfer accuracy coefficient (TAC) is proposed to characterize the performance of the model transfer. The TAC of samples is defined as:

(4) $$TAC=\frac{\left|R_{xy}\right|}{{∥x-y∥}_{2}}$$

(5) $$R_{xy}=\frac{\sum_{i=1}^{n}\left(x_{i}-\bar{x}\right)\left(y_{i}-\bar{y}\right)}{\sqrt{\sum_{i=1}^{n}{\left(x_{i}-\bar{x}\right)}^{2}\sum_{i=1}^{n}{\left(y_{i}-\bar{y}\right)}^{2}}}$$

(6) $$\bar{x}=\frac{\left(\sum_{i=1}^{n}x_{i}\right)}{n},\bar{y}=\frac{\left(\sum_{i=1}^{n}y_{i}\right)}{n},i=1,2,\ldots ,n$$

where *X* represents the spectrum of a master sample, *Y* represents the spectrum of a slave sample, $R_{xy}$ represents the correlation coefficient between the master sample and the slave sample and *n* represents the number of spectral wavelength points. The larger the transfer accuracy coefficient is, the better the model transfer effect is.

Firstly, all sample sets were randomly divided at a proportion of 60%, 20% and 20% into a calibration set, validation set and test set. Then, the objective function of the model transfer algorithm based on an autoencoder was trained and solved by using the steps of the training set and Bayesian optimization algorithm, and the performance of the model was tested by using the transfer accuracy coefficient information of the model calculated by the verification set. Finally, the test set was used to test the performance of the trained algorithm model. To sum it up, the model transfer algorithm based on an autoencoder is shown in Algorithm 1.
**Algorithm 1:** Model transfer algorithm based on an autoencoder**Input:** The master spectrometer data *X*, slave spectrometer data *Y*;**Output:** The reconstructed data after model transfer from the instrument;**1****Main procedure****2**Bias *B* and weight *W* in the autoencoder neural network of the master and slave instrument are randomly initialized. The parameters such as training times and learning rate of the model are set;**3**Compute the hidden layer variables $H_{x}$ and $H_{y}$, calculate the optimization objective function and optimize the whole model;**4**Optimized the hyperparameters of the autoencoder according to the Bayesian optimization algorithm, and the bias *B* and weight *W* were updated continuously by the gradient descent method until the optimal objective function value was found;**5**Save the slave encoder and the master decoder models to form the model transfer structure based on the improved autoencoder.

## 3. Results and Discussion

### 3.1. Experimental Data Set

**Figure 4:**
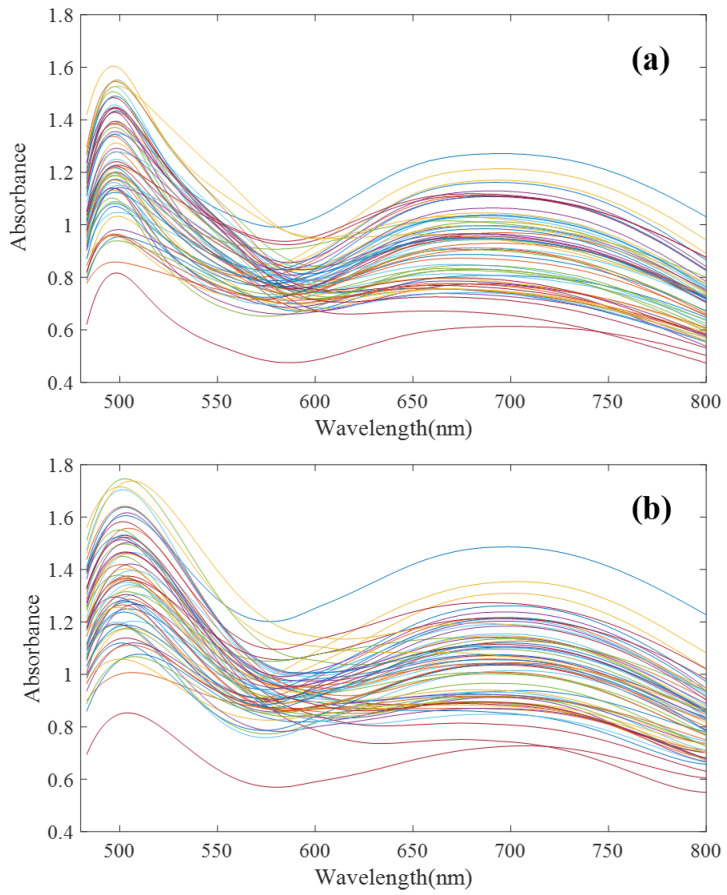
Ultraviolet Visible (UV-Vis) spectra of 64 groups of Cu, Co and Fe mixed solutions. (**a**) T9 spectrometer; (**b**) ATP2000 spectrometer (after denoising).

To verify the effectiveness of the proposed algorithm, in this paper, 64 groups of mixed solutions of copper, cobalt and iron under high zinc background were designed and configured according to an orthogonal test. Among them, zinc ion concentration was 33 g/L, and copper, cobalt, and iron ion concentration range was 2–16 mg/L. The T9 UV-Vis dual-beam spectrometer (Persee Instrument Co., Ltd., Beijing, China) and ATP2000 micro-spectrometer (Optosky Photonics Co., Ltd., Xiamen, China) were used to scan the spectral curves of 64 groups of samples at 480–800 nm. The spectral scanning results are shown in Figure 4.

To verify the necessity of spectral model transfer between the micro-spectrometer and traditional large spectrometer, the UV-Vis spectra of the solution with the same ion species and concentration were measured by using ATP2000 and T9 conventional spectrometers, respectively. The principal component score spatial distribution method was used to test the applicability of the model. It mainly used principal component analysis (PCA) to obtain the scores of samples in different principal components, then obtained the distribution map of the scores through calculation and then used the distribution map of the principal component score to judge the applicability of the model.

**Figure 5:**
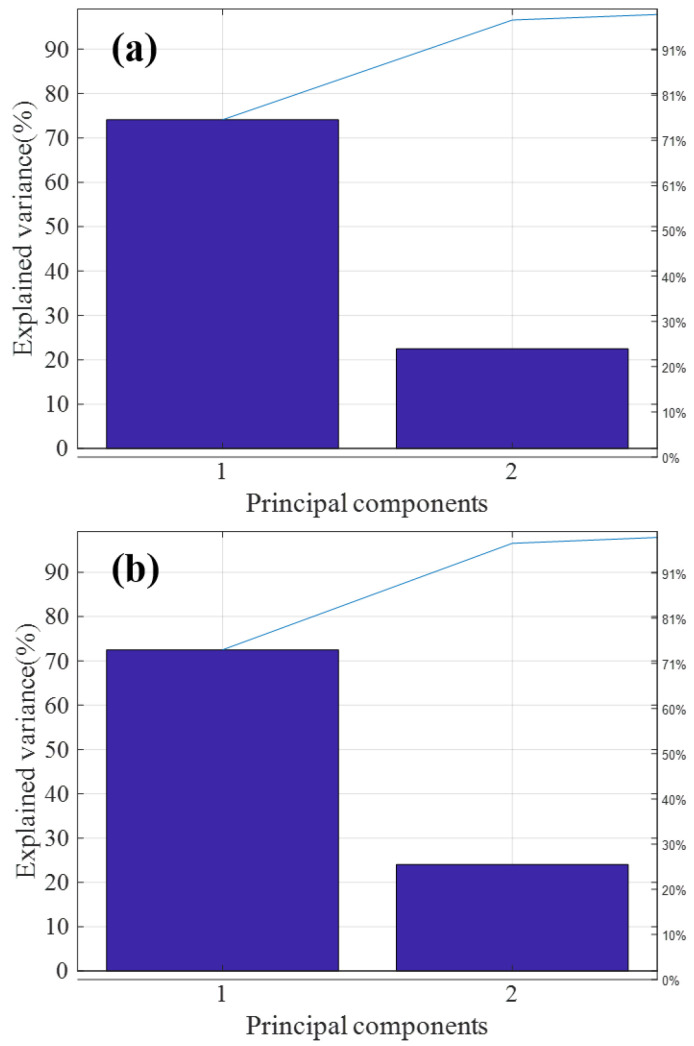
Principal component analysis (PCA) feature extraction of T9 and ATP2000 samples. (**a**) PCA feature extraction of the master spectrometer T9; (**b**) PCA feature extraction of the slave spectrometer ATP2000.

Figure 5a,b are the T9 and ATP2000 pareto graphs obtained after principal component analysis, respectively. The variance of the first two principal components of both was more than 95% of the total variance, so most of the information of the sample is generally reflected in the first and second principal components of the sample. Therefore, in this paper, the applicability of the model is judged by calculating the spatial distribution of the first and second principal component scores.

**Figure 6:**
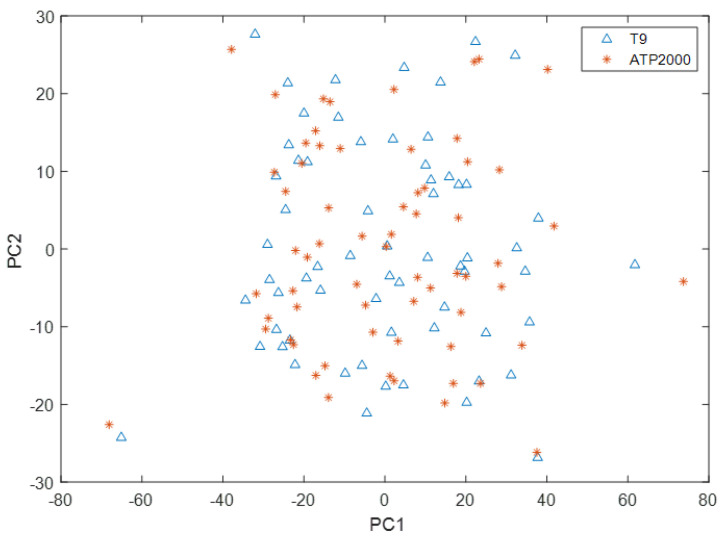
Spatial distribution of principal component scores of T9 and ATP2000 samples.

As can be seen from Figure 6, the scoring space of the first principal component and the second principal component of the T9 sample only partially overlapped with that of the ATP2000 sample, which did not completely cover the scoring space of the sample to be tested. Therefore, there is a great difference between the UV-Vis spectral data measured by the two UV-Vis spectrometers. The UV-Vis spectral data measured by T9 cannot directly build models to predict the ion concentration of samples measured by ATP2000. Therefore, it is necessary to solve this problem through model transfer.

### 3.2. Model Transfer Evaluation and Sample Number Selection

For model transfer, the fundamental purpose is to make the measurement under the different instrument condition of the same sample as close as possible, so as to ensure the multivariate calibration model can directly predict results. Set the $X=\{x_{1},x_{2},\ldots ,x_{m}\}$ as the master sample, $Y=\{y_{1},y_{2},\ldots ,y_{m}\}$ as the slave sample. The m is the number of samples.

For the whole test set, we used the average transfer accuracy coefficient $TAC_{ave}$ and the minimum transfer accuracy coefficient $TAC_{min}$ to evaluate the model transfer algorithm. The average transfer accuracy coefficient represents the overall performance of model transfer, and the minimum transfer accuracy coefficient represents the maximum possible transfer risk of model transfer, where $TAC_{ave}$ and $TAC_{min}$ are defined as:

(7) $$TAC_{ave}=\frac{1}{m}\sum_{i=1}^{m}TAC_{i}$$

(8) $$TAC_{\min}=\frac{1}{\min(TAC_{i})}$$

**Figure 7:**
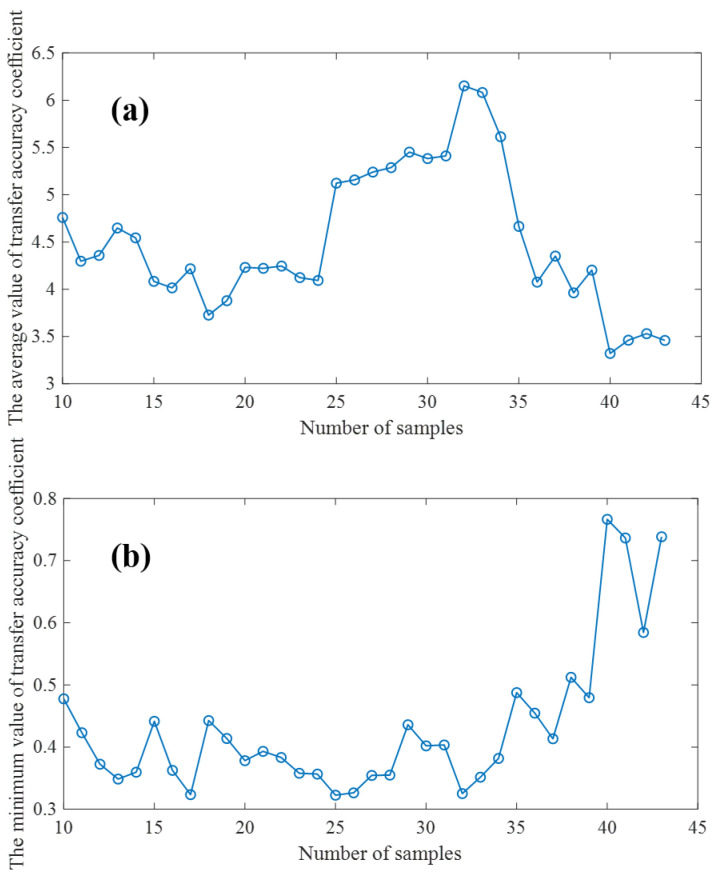
(**a**): Effects of transfer sample size on $TAC_{ave}$ (**b**): Effects of transfer sample size on $TAC_{min}$.

Different model transfer algorithms require different numbers of samples to be transferred, and there are slight differences in the methods used to select the transfer samples [29]. A more perfect model transfer algorithm needs fewer transfer samples to achieve good transfer performance. In order to quantitatively evaluate the model transfer performance, we first fixed all the parameters of the model transfer algorithm based on an autoencoder, and then adopted the K/S algorithm [30] to select the number of transfer samples of the proposed model transfer algorithm. The larger the average transfer precision coefficient and the smaller the minimum transfer precision coefficient of the test samples are, the better the model transfer effect is. Therefore, the optimal transfer sample number should be the number of samples at which $TAC_{ave}$ is large and $TAC_{min}$ is small simultaneously. In order to ensure the robustness and accuracy of the transfer model, we should not use too few samples for model training, and at the same time, we should ensure enough samples are used as verification sets. Therefore, when verifying the effect of the number of transferred samples on $TAC_{ave}$ and $TAC_{min}$, the range of samples is set from 10 to 43. As shown in Figure 7, under the condition that the number of transferred samples is as small a as possible and $TAC_{ave}$ as large as possible, as small $TAC_{min}$ as possible, the number of transferred samples should be 32.

**Figure 8:**
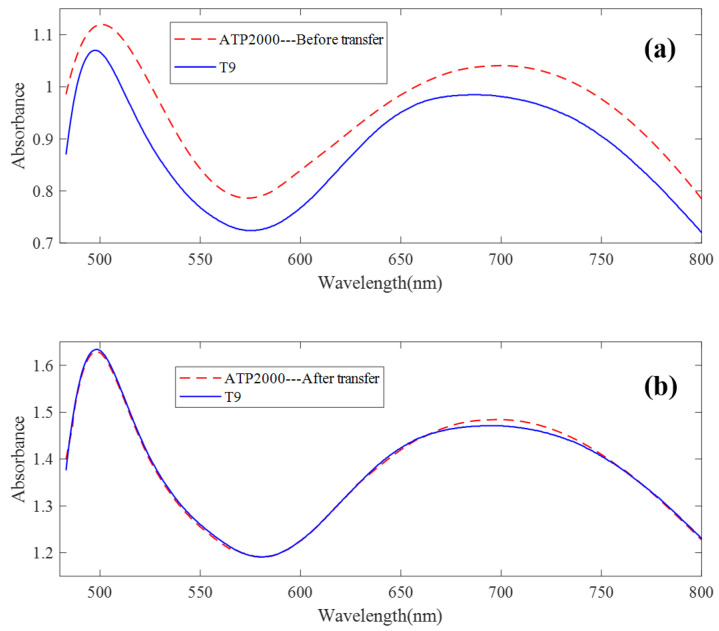
The spectral transfer result of the method. (**a**) The difference in spectral absorbance between the two instruments when measuring the same sample. (**b**) The spectrum measured by the slave instrument and the master spectrum after correction.

The UV-Vis spectrum correction model was established by a model transfer algorithm based on an autoencoder. It can be seen from Figure 4 that there are great differences between ATP2000 and T9 spectra of 64 groups of samples scanned by two different spectrometers. Figure 8a shows the difference in spectral absorbance between the two instruments ATP2000 and T9 when measuring the same sample. It can be seen from the figure that the two spectral curves present an obvious state of baseline drift, and the difference at each wavelength point is nonlinear, especially at the peaks and troughs of the spectrum. It can be seen from the two characteristic peaks that the two absorbance curves also have the phenomenon of wavelength point drift. After the transfer of the model proposed in this paper, the spectrum measured by the slave instrument is basically coincident with the master spectrum after correction. The phenomenon of wavelength point shift is basically eliminated, as shown in Figure 8b.

In order to verify the effectiveness of the proposed algorithm and compare it with DS and PDS algorithms, the average and minimum transfer accuracy coefficients of the three model transfer algorithms were, respectively, calculated using 32 validation sets samples, as shown in Table 1.

**Figure 9:**
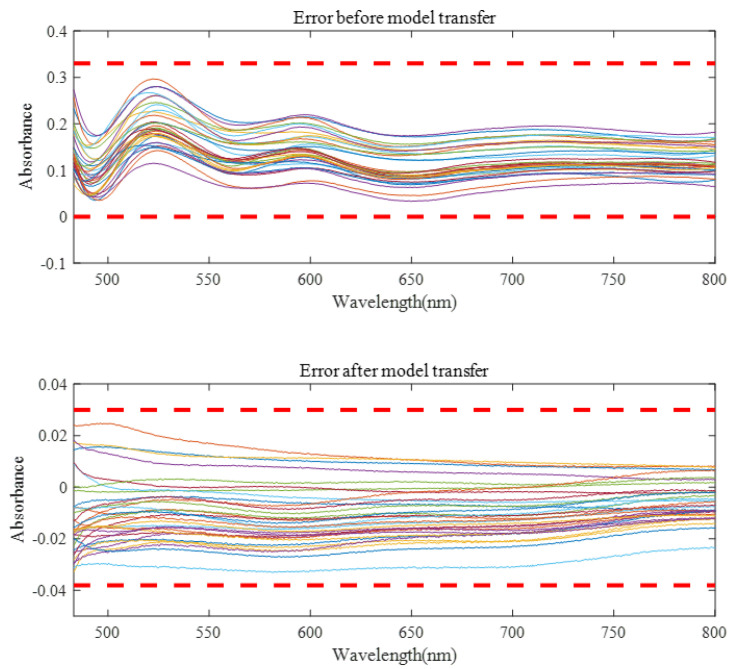
The spectral errors of the model were verified before and after spectral transfer.

Before model transfer, there was a big difference in measured spectra between instruments. After spectral transfer through the three model transfer methods, the average transfer accuracy coefficient between the master and the slave instrument was improved, while the minimum transfer accuracy coefficient was reduced. As can be seen from Figure 9, the spectral error of 32 validation set samples before model transfer is about 0–0.3, and the spectral difference can be controlled within −0.03–0.03 after model transfer of the algorithm in this paper. It can be seen from Table 1 that the proposed algorithm has the best model transfer performance. Compared with the DS algorithm and PDS algorithm, the average transfer accuracy coefficient increased by 45.11.% and 22.38%, respectively, and the minimum transfer accuracy coefficient decreased by 44.32% and 36.84%, respectively.

To further verify the effectiveness of the algorithm, compared with an algorithm based on the DS and PDS, the transfer model was established by using a multivariate calibration model of least squares to predict concentration. As shown in Table 2, the root mean square error of prediction results was used to validate the results.

According to Table 2, huge deviations occurred in the prediction of sample data by using the partial least squares (PLS) regression model established before model transfer, indicating that the model established on the master instrument had basically become completely invalid. After three model transfer algorithms, the predicted mean square error decreased. However, the DS algorithm adopted all the wavelength points to establish the transfer model. In the high-dimensional spectral background, the wide modeling range is very likely to cause the transfer model error. Although PDS establishes a window near a certain wavelength point and builds the transfer model in a small range, the spectral data collected by spectrometers ATP2000 and T9 will also have offset due to the different spectral resolution, and the window size will also affect the performance of the model. It can be seen from Table 2 that the algorithm proposed in this paper has the best prediction ability of ion concentration compared with the comparison algorithm. Compared with the DS algorithm and PDS algorithm, the root mean square error of Cu${}^{2+}$ prediction was reduced by 92.88% and 75%, respectively. The prediction error of Co${}^{2+}$ was reduced by 87.37% and 84.47%, respectively. The prediction error of Fe${}^{2+}$ was decreased by 67.81% and 65.94%. The prediction accuracy of the ion concentration was greatly improved.

## 4. Conclusions

For spectral difference between a traditional desktop spectrometer and micro-spectrometer caused by hardware, this paper proposes a model transfer algorithm based on the improved autoencoder. Firstly, two autoencoders are used to train the spectral data of the master and slave instrument, respectively. Then, the two encoder is added with hidden variable constraint conditions, which makes the two hidden variables equal. A Bayesian optimization algorithm and transfer accuracy coefficient are proposed to optimize the model. Finally, the hidden variables from the instrument are obtained through the decoder of the master instrument to obtain the final reconstruction data, so as to complete the process of model transfer. The experimental results show that the average transfer accuracy coefficient of the proposed algorithm is improved by 45.11% and 22.38%, and the minimum transfer accuracy coefficient is reduced by 44.32% and 36.84% compared with the DS algorithm and PDS algorithm, respectively. Therefore, the proposed method can reduce the spectrum difference between the micro-spectrometer and the traditional large spectrometer and realize the data sharing between different instruments.

## Figures and Tables

**Table 1:** Comparison of transfer performance of three model transfer algorithms.

Method	$\mathrm{TA}C_{\mathrm{ave}}$	$\mathrm{TA}C_{\min}$
Before model transfer	3.5727	0.8211
DS	4.2391	0.5841
PDS	5.0269	0.5149
Proposed method	6.1517	0.3252

**Table 2:** Comparison of transfer performance of three model transfer algorithms of the test set.

RMSE	Cu${}^{2+}$	Co${}^{2+}$	Fe${}^{2+}$
Before model transfer	0.426	0.397	0.443
DS	0.295	0.198	0.146
PDS	0.084	0.161	0.138
Proposed method	0.021	0.025	0.047

## Data Availability

The data presented in this study are not publicly available at this time, but may be obtained upon reasonable request from the authors.
